# Cronkhite‒Canada syndrome as inflammatory hamartomatous polyposis: new evidence from whole transcriptome sequencing of colonic polyps

**DOI:** 10.1186/s13023-024-03038-8

**Published:** 2024-02-01

**Authors:** Shuang Liu, Yunfei Zhi, Runfeng Zhang, Yan You, Wen You, Qiushi Xu, Jingnan Li, Ji Li

**Affiliations:** 1 Department of Allergy, Chinese Academy of Medical Sciences, Peking Union Medical College Hospital, 100730 Beijing, People’s Republic of China; 2 Department of Gastroenterology, Chinese Academy of Medical Sciences, Peking Union Medical College Hospital, Beijing, 100730 People’s Republic of China; 3 Department of Internal Medicine, Chinese Academy of Medical Sciences, Peking Union Medical College Hospital, 100730 Beijing, China; 4 Department of Pathology, Chinese Academy of Medical Sciences, Peking Union Medical College Hospital, 100730 Beijing, People’s Republic of China; 5 Department of Gastroenterology, First Affiliated Hospital of Nanjing Medical University, 210029 Nanjing, People’s Republic of China

**Keywords:** Cronkhite‒Canada syndrome, Whole transcriptome sequencing, Colon hamartomatous polyps

## Abstract

**Background:**

Cronkhite-Canada syndrome (CCS) is a rare, nonhereditary disease characterized by diffuse gastrointestinal polyposis and ectodermal abnormalities. Although it has been proposed to be a chronic inflammatory condition, direct evidence of its pathogenesis is lacking. This study aims to investigate the pathophysiology of CCS by analyzing transcriptomic changes in the colonic microenvironment.

**Methods:**

Next-generation sequencing-based genome-wide transcriptional profiling was performed on colonic hamartomatous polyps from four CCS patients and normal colonic mucosa from four healthy volunteers. Analyses of differential expression and multiple enrichment analyses were conducted from the molecular level to the cellular level. Quantitative real-time PCR (qRT-PCR) was carried out to validate the sequencing accuracy in samples from six CCS patients and six healthy volunteers.

**Results:**

A total of 543 differentially expressed genes were identified, including an abundance of CC- and CXC-chemokines. Innate immune response-related pathways and processes, such as leukocyte chemotaxis, cytokine production, IL-17, TNF, IL-1 and NF-kB signaling pathways, were prominently enhanced in CCS colonic polyps. Upregulation of wound healing, epithelial-mesenchymal transition, Wnt, and PI3K-Akt signaling pathways were also observed. Enrichment analyses at different levels identified extracellular structure disorganization, dysfunction of the gut mucosal barrier, and increased angiogenesis. Validation by qRT-PCR confirmed increased expression of the *LCN2*, *IL1B*, *CXCL1*, and *CXCL3* genes in CCS colonic polyps.

**Conclusions:**

This case-control whole transcriptome analysis of active CCS colonic hamartomatous polyps revealed intricate molecular pathways, emphasizing the role of the innate immune response, extracellular matrix disorganization, inflammatory cell infiltration, increased angiogenesis, and potential epithelial to mesenchymal transition. These findings supports CCS as a chronic inflammatory condition and sheds light on potential therapeutic targets, paving the way for more effective and personalized management of CCS in the future.

**Supplementary Information:**

The online version contains supplementary material available at 10.1186/s13023-024-03038-8.

## Background

Cronkhite‒Canada syndrome (CCS) is a rare, nonhereditary disease characterized by diffuse gastrointestinal (GI) polyposis, presenting with refractory diarrhea, abdominal pain, and anorexia, alongside an ectodermal triad of alopecia, nail changes and hyperpigmentation [1, 2]. Since the first case was reported in 1955, over 500 CCS cases have been documented until 2014 [3]. The prevalence of CCS in Japan is 3.7/1,000,000, while epidemiological data in other parts of the world are still unavailable [4]. CCS is a generally elderly-onset condition (average onset age of 59), and over 80% of patients get their diagnosis after 50 years old [2]. The typical initial endoscopic findings of CCS include multiple polyps on the background of diffuse mucosal hyperemia and edema, which frequently affect the entire GI tract except the esophagus. Histologically, CCS features hamartomatous polyps with congestion and chronic inflammation of the lamina propria and submucosa [5–7].

To date, the pathogenesis of CCS remains unclear [3]. It has been hypothesized that CCS is a chronic inflammatory condition associated with autoimmune mechanisms and intestinal mucosal damage. Several studies support this hypothesis from the perspectives of clinical association with autoimmune indicators, histological infiltration of IgG4-positive plasma cells, and treatment responses to immune modulating therapies (corticosteroids, immunosuppressants, and tumor necrosis factor inhibitors); however, direct evidence is still lacking [7–10]. Approximately 80% of CCS patients respond effectively to glucocorticoids and other immunosuppressive therapies (such as azathioprine), achieving clinical remission and polyp disappearance to varying degrees [5]. While classical CCS polyps are considered non-neoplastic, 15% to 25% of patients have been documented to have GI neoplasia at diagnosis, and up to 40% have adenomas or adenomatous lesions at different stages [3, 11, 12]. Despite an improvement in prognosis over the past 40 years [2, 5, 13, 14], understanding the pathogenesis remains pivotal for enhanced disease management.

Whole exosome sequencing (WES) and whole transcriptome sequencing (WTS) serve as crucial tools for investigating rare diseases. In the MASTER (Molecularly Aided Stratification for Tumor Eradication Research) study, WTS provided diagnostic and therapeutic guidance for patients with rare cancers [15]. The advantages of WTS allow to better understand rare diseases by tracking and revealing the causatives of different diseases and identifying abnormalities at the tissue level [16]. In our previous study and several other case reports, WES was performed to detect germline mutations in CCS patients, in which potential roles of innate immune responses and glycosylation were highlighted, while pathogenic germline variants in hallmark genes of classical hereditary hamartomatous polyposis syndrome (HPS) were not found [12, 17, 18]. A recent study exploring the WTS characteristics indicated increased *INHBA* mRNA and protein expression in the CCS gastric polyp [19].

To delve deeper into the CCS pathogenesis, we performed WTS on colonic hamartomatous polyps from active-phase CCS and conducted quantitative real-time PCR to validate the results.

## Methods

### Clinical sample collection and preparation

Biopsy samples for transcriptional analysis and validation via quantitative real-time PCR (qRT-PCR) were collected from patients in the active phase of CCS (aCCS) during routine clinical care or from matched healthy volunteers (HV) attending routine colonoscopy screening between December 2018 and January 2020.

Endoscopic evaluation and biopsy procedures for CCS patients were conducted by an experienced gastroenterologist. All samples were examined and diagnosed as CCS colonic hamartoma polyps by an expert GI pathologist, who was blinded to the relevant clinical and endoscopic information. Typical pathological features of hamartomatous polyps included edematous lamina propria, cystically dilated and distorted glands and mononuclear inflammatory cell infiltration. Representative endoscopic and histopathological features of which were shown in Fig. 2A, B, respectively.

**Fig. 1:**
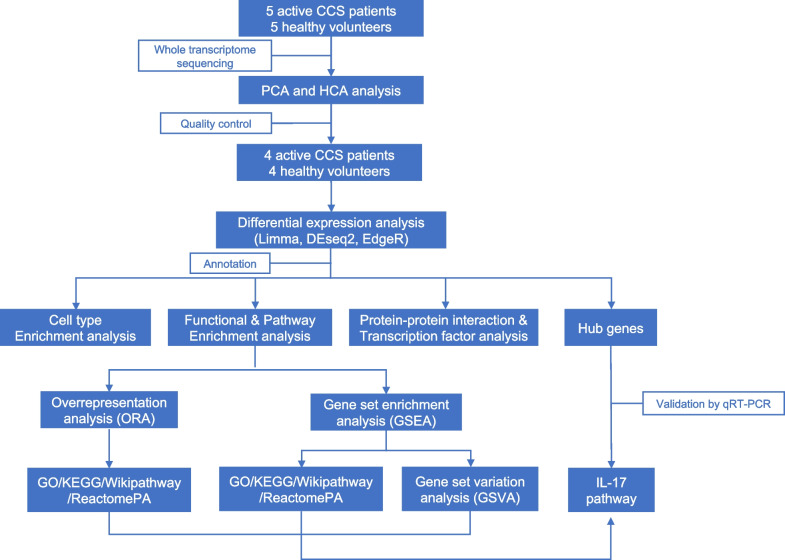
Flowchart of this study. CCS, Cronkhite-Canada syndrome. HCA, hierarchical cluster analysis. PCA, principal component analysis. qRT-PCR: quantitative real-time PCR

The baseline information of aCCS patients and HVs is shown in Table 1 (for the transcriptional analysis group) and Table S1 (for the validation group), respectively. The workflow is illustrated in Fig. 1.

**Table 1:** Baseline characteristics of patients and healthy volunteers in transcriptional analysis group

	Patients in the positive phase of CCS (pCCS)						Healthy Volunteers (HV or Control)				Consistency of Biopsy	
	Age	Gender	Tissue	Proximodistal histopathology	Disease status	Glucocorticoid treatment		Age	Gender	Proximodistal histopathology	Time	Location
CCS1	63	Female	Colon	(Ascending colon) mild acute and chronic inflammation	Positive	No	Control1	53	Female	Healthy colon	Yes	Yes
CCS2	56	Female	Colon	(Descending colon) significant acute and chronic inflammation	Positive	No	Control2	60	Male	Healthy colon	Yes	Yes
CCS3	61	Female	Colon	(Descending colon) significant acute and chronic inflammation	Positive	Yes	Control3	83	Female	Healthy colon	Yes	Yes
CCS4	61	Male	Colon	(Terminal ileum, hepatic flexure, transverse colon, splenic flexure) consistent with the intestinal changes seen in CCS	Positive	Yes	Control4	55	Male	Healthy colon	Yes	Yes
CCS5	46	Male	Colon	(Ascending colon) markedly mild acute inflammation and chronic inflammation; (Large polyps in transverse and ascending colon) tubular adenoma of the gland	Positive	No	Control5	48	Male	Healthy colon	Yes	Yes

The positive phase was defined as the occurrence of typical clinical symptoms (including diarrhea and ectodermal changes) and endoscopically visible diffuse or multiple polyps in the gastrointestinal tract. Healthy volunteers were required to have no previous inflammatory disease of the digestive tract diagnosed, normal appearance of colonic mucosa at endoscopy and no multiple polyps of the digestive tract

This study was approved by the Ethics Committee of Peking Union Medical College Hospital (I-22PJ1077), and informed consent was obtained from all subjects in accordance with the Declaration of Helsinki.

### RNA extraction and sequencing library construction

Total RNA of biopsy samples was extracted using TRIzol reagent (Life Technologies, USA). The purity and integrity of total RNA were assessed by an Agilent 2100 bioanalyzer (Agilent Technologies, USA). Then, the Illumina complementary DNA (cDNA) library was constructed using the TruSeq RNA Library Prep Kit (Illumina, USA). The cDNA library underwent end repair, addition of a single ʻAʼ base, ligation of the adapters, purification, and enrichment according to the manufacturer’s instructions. The insert size of the cDNA library was assessed using the ABI StepOnePlus Real-Time PCR System (Thermo Fisher Scientific, USA) and Agilent 2100 Bioanalyzer (Agilent Technologies, USA). Subsequently, RNA sequencing was conducted on the HiSeq X Ten platform (Illumina, USA) to generate 150 bp paired-end reads. Raw data obtained in reads in fastq format were trimmed with Cutadapt software version 1.14 (https://github.com/marcelm/cutadapt/). The reads were mapped to the GRCh38 reference human genome via Hisat2 software version 2.0 (https://daehwankimlab.github.io/hisat2) and quantified as the fragments per kilobase of exon model per million mapped reads (FPKM) value via StringTie software version 1.3 (https://ccb.jhu.edu/software/).

## Data analysis

### Quality control

Principal component analysis (PCA) is a commonly used multivariate statistical analysis method with unsupervised learning that can effectively reduce the dimensionality of multivariate data and demonstrate individual differences and intergroup relationships of samples [20]. Hierarchical cluster analysis (HCA) is an unsupervised clustering method [21] that uses a linkage-based tree diagram to evaluate and visualize data similarity between samples. To ensure robust analysis, PCA and HCA were utilized to evaluate the inter- and intragroup differences between the aCCS and HV groups. Samples displaying high heterogeneity were excluded.

### Differential expression analysis and gene annotation

Differential expression analysis between aCCS and HV groups based on the expression count matrix was processed by three R packages, i.e., *DESeq2* [22], *edgeR* [23], and *limma* [24]. The genes that simultaneously met the threshold of |log2 Fold Change|> 2 and *p*-value < 0.01 by *DESeq2*, *edgeR*, and *limma* were identified as convincing differentially expressed genes (DEGs). The fold changes and *p*-values calculated by *DeSeq2* were extracted for subsequent analyses. Annotation was performed by the R package *org.Hs.eg.db*. DisGeNet, a discovery platform containing collections of genes related to human diseases, to examine whether the selected samples could reflect disease characteristics [25].

### Functional and pathway enrichment analyses

To interpret the transcriptome sequencing data comprehensively, functional and pathway enrichment analyses were performed using diverse database resources, including Kyoto Encyclopedia of Genes and Genomes (KEGG) pathways [26], Gene Ontology (GO) database [27], WikiPathways database [28], and ReactomePA database [29]. Two principal approaches, over representation analysis (ORA) and gene set enrichment analysis (GSEA), were adopted to process the analyses [30]. The difference between the ORA approach and the GSEA approach lies in the entering items for analyses. The ORA approach is based on the DEGs to provide a representative profile, while the GSEA approach was based on the entire gene set to obtain a holistic view of the data.

Specifically, the ORA approach utilized lists of upregulated and downregulated DEGs to identify enriched functional modules or pathways within the GO, KEGG, ReactomePA, and WikiPathways databases. On the other hand, the GSEA approach, analyzed the entire gene set expression data based on the same databases. Additionally, the hallmark gene sets from the Molecular Signatures Database (https://www.gsea-msigdb.org) were downloaded to construct a pathway-centric view based on the entire expression data, using the gene set variation analysis (GSVA), which can be regarded as a particular method of the GSEA approach [31].

In essence, we utilized a repertoire of databases to extract nuanced insights into the functional landscape of the transcriptome. This comprehensive analytical framework ensures a thorough exploration of the molecular intricacies underpinning our findings. The abovementioned analyses were performed with the R package *ClusterProfiler* [32].

### Protein‒protein interaction and transcription factor analysis

The STRING database (https://string-db.org) was used to perform protein‒protein interaction (PPI) analysis with the upregulated and downregulated DEGs. The upregulated and downregulated DEGs were entered into STRING to calculate the PPI score. Cytoscape (https://cytoscape.org) was used to visualize the data downloaded from the STRING database. To balance sensitivity and specificity, hub genes were selected using the following steps: (1) the top 30 upregulated or downregulated DEGs were filtered by 11 different topology algorithms embedded in the cytoHubba plugin [33]; (2) the 20 most frequently occurring genes were selected to build the hub gene network. KEGG analysis was performed to identify the hub pathways. The transcription factor (TF)–target interaction (TRRUST2) database (https://www.grnpedia.org/trrust) was employed to predict the enriched regulons.

### Cell type enrichment analysis

Because tissues are a complex milieu composed of different cell types, the result of WTS can be seen as the sum of the gene expression of various cells. Microenvironment Cell Populations-counter (MCP-counter) is a robust quantification method for heterogeneous tissues to perform cell type enrichment analysis via gene expression data [34]. In this study, custom cell type enrichment analysis of epithelial cells and fibroblasts was also performed via GSVA using gene sets obtained from published studies [35]. The Mann–Whitney U test was applied to calculate the significance of the difference in the distributions between groups. The threshold was set at *p*-value < 0.05 with the consideration of both sample size and *p*-value distribution. Intercellular correlation and intercellular hub-gene correlation analyses were performed via ggcorplot.

### RNA extraction and quantitative real-time PCR

Total RNA was extracted with TRIzol (10,296,010, Invitrogen) and converted to cDNA using Prime Script RT Master Mix (RR036A, Takara Bio). The remaining cDNA samples after RNA sequencing were returned to us for further use. All five CCS samples and three HV samples (two included in the final analysis) were adequate for subsequent qRT-PCR experiments. We further collected another one CCS sample and three HV biopsy samples for RNA extraction and cDNA preparation. qRT-PCR was performed on a CFX96 real-time PCR system (Bio-Rad, USA) using Taq Pro Universal SYBR qRT-PCR Master Mix Q712 (Vazyme, China). Specific primers for each gene were designed via Primer Premier version 5.0 (Premier, Canada). The relative RNA expression level was normalized to GAPDH messenger RNA according to the 2^−ΔΔCt^ calculation method. The primer sequences are shown in Table S2.

### Statistical analysis

Statistical analyses were processed by GraphPad Prism 9 (https://www.graphpad.com) and R version 4.1 (https://www.R-project.org/). Unless otherwise specified, a Benjamini–Hochberg corrected *p*-value < 0.05 was considered the threshold for enrichment analysis. For nonparametric tests, a two-tailed *p*-value < 0.05 was considered statistically significant.

## Results

### Baseline information and quality control

As detailed in Table 1, colonic biopsy samples were obtained from five CCS patients and five healthy volunteers. WTS generate gene expression profiles, which were converted into count matrix and FPKM matrix format. PCA and HCA analysis based on the count matrix identified sample CCS5 as an outlier (Fig. S1A–C). Because PCA and HCA revealed clear separation between aCCS samples and controls after removing CCS5 and its paired sample (Fig. S1D–F), we excluded them in the subsequent analysis. CCS5 exhibited distinct clinical characteristics, being younger at disease onset (46 years old), showing an unsatisfactory response to glucocorticoid treatment, and eventually succumbing to gastrointestinal bleeding, unlike the other patients who were older at disease onset and achieved clinical remission as well as polyp regression after glucocorticoid treatment.

**Fig. 2:**
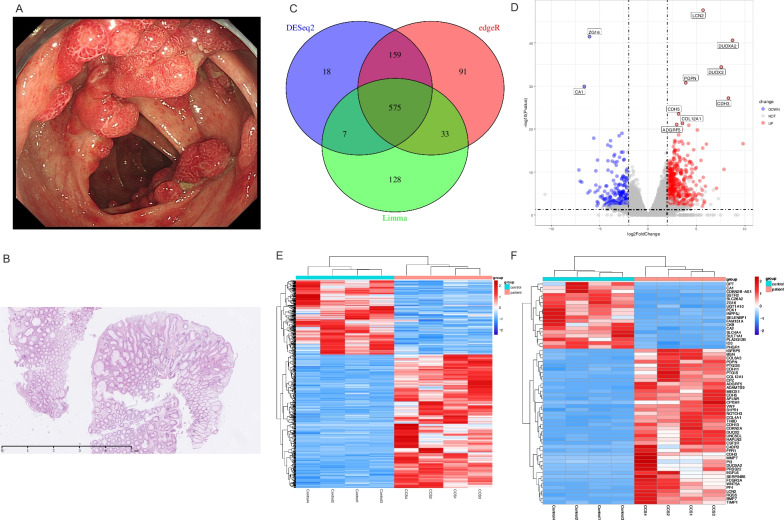
Overview of studied sequencing samples. **A** Classic endoscopic figure of colon polyps in aCCS patients. **B** Classic histopathologic figure of colon polyps in aCCS patients. **C** The Venn diagram shows the overlap and differences between the results obtained with three different methods (DESeq2, edgeR, Limma); **D** Volcano plot showed DEGs between aCCS and controls; **E** Heatmap of all the DEGs identified by three methods among all the samples; **F** Heatmap of top 60 DEGs identified by three methods in analysis. aCCS, in the active phase of Cronkhite-Canada Syndrome

A total of 21,968 genes that were effectively expressed in over 25% of the samples were included in the differential analysis pipeline. Finally, 576 differentially expressed genes (including 373 upregulated and 203 downregulated) were identified as convincing DEGs, and more than 80% of the effectively expressed genes were successfully annotated, including 543 (94%) DEGs (Fig. 2C–F).

To confirm that the DEGs can reflect the features of CCS, disease enrichment analysis using the DisGeNet database was processed with the DEGs to examine whether the enrichment results were in accordance with the clinical knowledge of CCS. Consequently, multiple disease gene sets associated with CCS were localized (Fig. S1G).

### Functional and pathway enrichment analyses

Enrichment analyses were performed using the ORA approach for DEGs and the GSEA approach for all genes. As detailed in the Methods section, DEGs and the entire gene set data were used for the ORA approach and the GSEA approach, respectively. Analyses were conducted based on multiple databases, including the GO, KEGG, ReactomePA, and WikiPathways databases. Subsequently, GSVA based on the hallmark gene set database was employed to provide a more comprehensive interpretation of the expression profile.

**Fig. 3:**
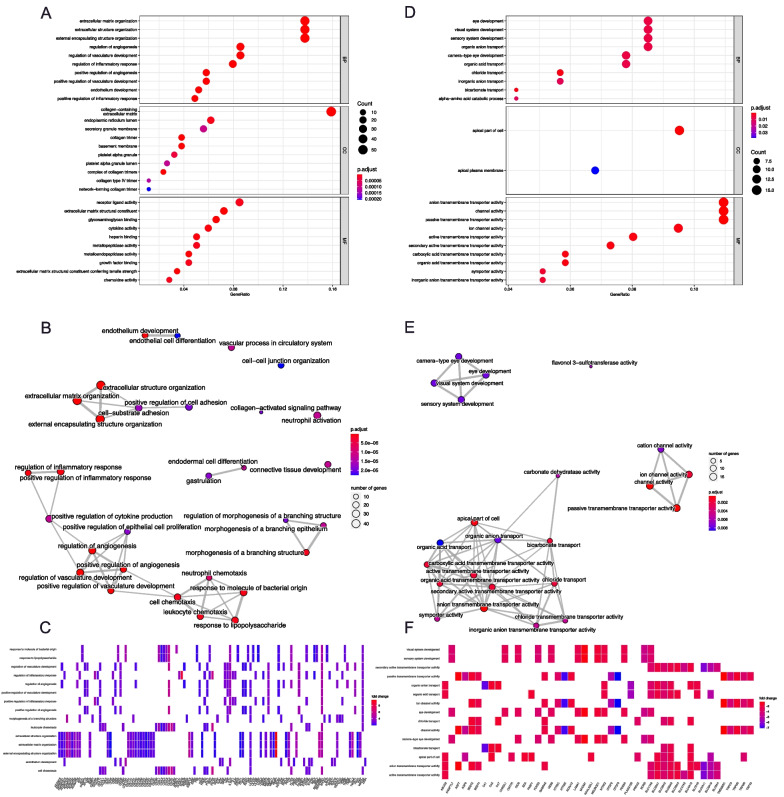
Results of GO ORA analyses. **A** Result of GO ORA analysis based on upregulated DEGs; **B** Clustering result of GO ORA analysis based on upregulated DEGs; **C** Heatmap showed the relationship between upregulated DEGs and upregulated pathways; **D** Result of GO ORA analysis based on downregulated DEGs; **E** Clustering result of GO ORA analysis based on downregulated DEGs; **F** Heatmap showed the relationship between downregulated DEGs and downregulated pathways. GO, gene ontology. ORA, over representation analysis. DEG, differentially expressed genes.

Enrichment analyses via the ORA approach provide information based on DEGs. The GO-based analyses for DEGs were clustered into different functional modules. For upregulated DEGs, regulation of extracellular matrix (ECM) organization, angiogenesis and inflammation response were significantly enriched, and multiple types of collagen-related pathways and expression of cytokines and cytokine receptors were involved as well (Fig. 3A, B). Gene-pathway heatmaps were drawn to visualize the relationship between different pathways and DEGs. As shown in Fig. 3C, matrix metalloproteinases (MMPs), A Disintegrin and Metalloproteinase domains (ADAMs), chemokines and chemokine receptors were prominent. For downregulated DEGs, channel activity- and anion transport-associated pathways were identified (Fig. 3D, E). The solute carrier family (SLC), transient receptor potential (TRP) and carbonic anhydrase family (CA) genes were frequently involved (Fig. 3F).

**Fig. 4:**
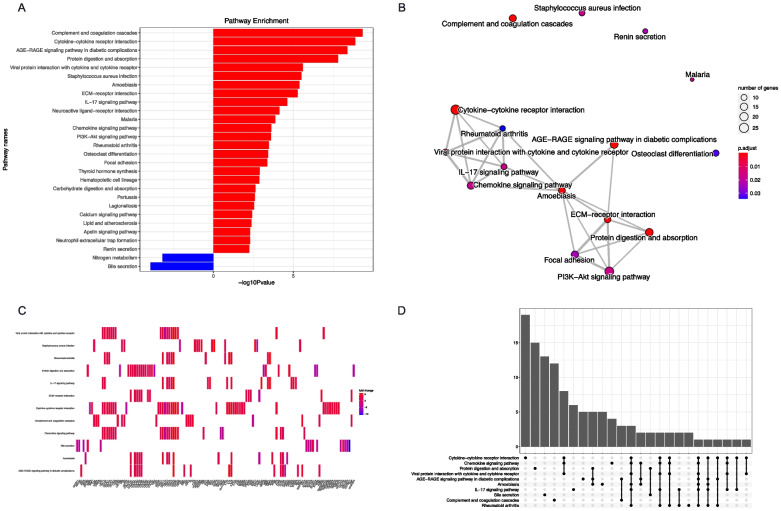
Results of KEGG ORA analyses. **A** Result of KEGG ORA analysis based on DEGs, x-axis indicated the -log10 value of *P*-value; **B** Clustering result of KEGG ORA analysis based on DEGs; **C** Heatmap showed the relationship between DEGs and pathways; **D** Upsetplot showed the relationship between different pathways, which indicating that IL-17 pathway was associated with multiple pathways. KEGG, Kyoto Encyclopedia of Genes and Genomes. ORA, over representation analysis. DEG, differentially expressed genes

The results of KEGG-based DEG enrichment analyses are shown in Fig. 4. Compared with GO-based enrichment, KEGG-based enrichment mapped to more specific signaling pathways including complement and coagulation cascades, the IL-17 signaling pathway, the PI3K-Akt signaling pathway, the AGE-RAGE signaling pathway in diabetic complications, neutrophil extracellular trap formation, etc. (Fig. 4A). The clustering results showed that the module consisting of immunity- and inflammation-related signaling pathways was prominently enriched (Fig. 4B). The gene-pathway heatmap based on KEGG analyses was similar to the GO-based result (Fig. 4C). An UpSet plot was drawn to visualize the overlaps between genes and pathways (Fig. 4D). The processes of protein digestion and absorption were highly enriched in upregulated DEGs, while nitrogen metabolism and bile secretion were enriched in downregulated DEGs.

To obtain further information on CCS, ReactomePA-based (Fig. S2A) and WikiPathways-based (Fig. S2B) enrichment based on DEGs was performed. The overall result was similar to the results of GO- and KEGG-based analyses; however, the WikiPathways enrichment analysis identified epithelial to mesenchymal transition (EMT) in the colorectal cancer pathway and the PPAR anti-inflammatory pathway with upregulated and downregulated DEGs, respectively.

**Fig. 5:**
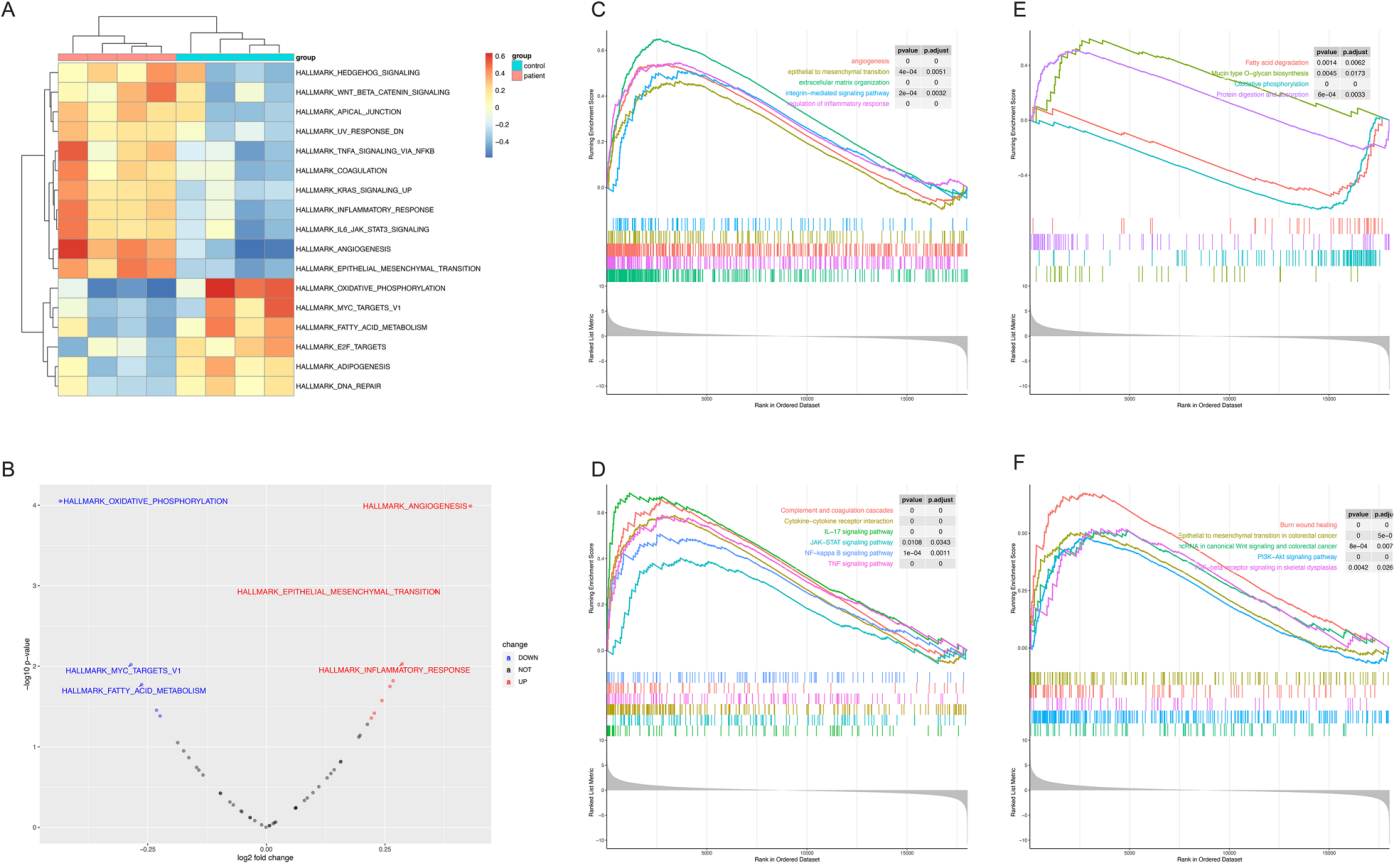
Results of GSVA analyses and GSEA plots for core pathways. **A** Heatmap showed the GSVA result based on Hallmark gene set. Pathways with *P*-value < 0.005 were shown in the heatmap; **B** Volcano plot showed distribution of pathways; **C** GSEA plot for the histology features of CCS colonic hamartomatous polyp; **D** GSEA plot for innate immune associated signaling pathways related to CCS; **E** GSEA plot for down-regulated and metabolism pathways in CCS; **F** GSEA plot for cancer-associated pathways regulated in CCS. GSVA, Gene Set Variation Analysis; GSEA, Gene Set Enrichment Analysis; CCS, Cronkhite-Canada Syndrome

Enrichment analyses with the GO database (Fig. S2C) or KEGG database (Fig. S2D) via the GSEA algorithm for all genes were similar to the ORA approach, which were consistent with the result based on ReactomePA and WikiPathways database (Fig. S2E, F). Considering that most abovementioned pathways were covered by the hallmark gene set, we subsequently performed GSVA analysis based on this gene set. As shown in Fig. 5A, B, pathways including angiogenesis, EMT, inflammatory response, IL6-JAK-STAT3 signaling pathway, KRAS signaling pathway, coagulation, and TNF-α signaling were highly upregulated. The abovementioned finding was consistent with previous analyses. Meanwhile, oxidative phosphorylation and the fatty acid metabolism pathway were downregulated. Of note, the MYC target gene set and E2F target gene set were downregulated in GSVA analysis, but MYC target gene set members were not on our DEG lists. In the E2F target gene set, *CDKN2A* was an upregulated DEG that was reported to negatively regulate the pRb-E2F pathway during the cell cycle [36] (Fig. 5A, B). Pathways relevant to certain pathophysiological processes were separately plotted (Fig. 5C–F) and addressed in the Discussion section.

The source data of the enrichment analysis are provided in Table S3.

### Protein‒protein interaction and transcription factor analysis

**Fig. 6:**
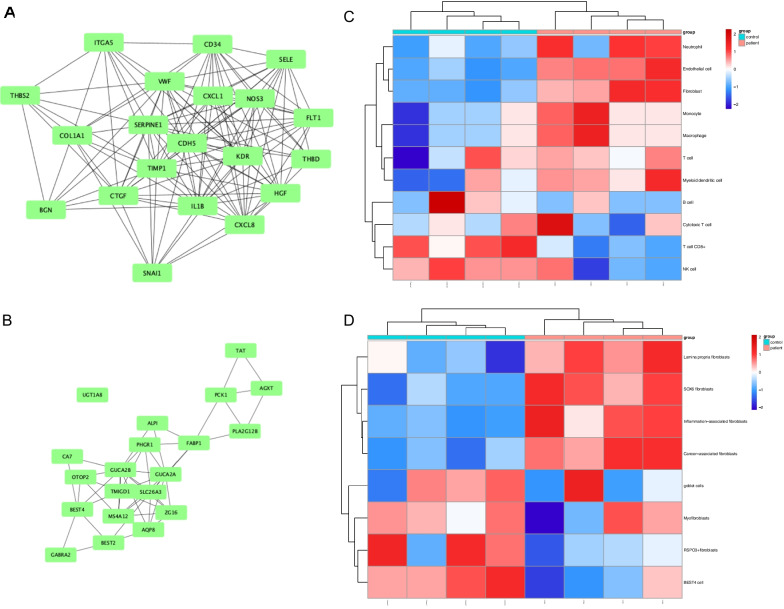
Results of PPI analyses and Cell Marker Enrichment analyses. **A** Upregulated hub genes; **B** Downregulated hub genes; **C** Heatmap showed the cell enrichment scores calculated by MCP-counter; **D** Heatmap showed the cell enrichment scores of fibroblasts and epithelial cells calculated by GSVA (The specific method is ‘ssgsea’). PPI, Protein–protein interaction

Separate hub gene networks for upregulated (Fig. 6A) and downregulated (Fig. 6B) genes were obtained from PPI and topology analysis. Correlation analyses were applied between the hub genes (Fig. S3A). Enrichment analysis of hub genes with the GO database or KEGG database via the ORA approach was consistent with the analysis of all DEGs (Fig. S3B, C).

Upregulated hub genes were highly associated with the regulation of inflammation, angiogenesis, ECM and hemostasis, which was consistent with the abovementioned enrichment analyses. Among the downregulated hub genes, *BEST4* and *OTOP2* were important cell markers for *BEST4* + cells, and *ZG16* and *BEST2* were goblet cell-specific [37]. The downregulation of these genes may indicate a decreasing tendency of these two cell types in aCCS. Cell marker enrichment analysis based on BEST4 + and goblet cell-specific gene sets was performed to verify our hypothesis (Fig. 6D).

Later, all DEGs and hub genes were entered into the TRRUST2 database to identify key transcription factors. The results of DEGs and hub genes were highly consistent, indicating that RELA, NFKB1, and SP1, the top 3 transcription factors shared in the analyses, may play certain roles in CCS. In addition, the STAT family (including STAT1, STAT3, and STAT6) was also identified by both analyses. The full results of the analyses are shown in Tables S3 and S4.

### Cell type enrichment analysis

The quantification of the absolute abundance of eight immune and two stromal cell populations was calculated via MCP-counter using the Mann–Whitney U test. Fibroblasts and endothelial cells were found to be significantly upregulated in aCCS, which was in accordance with the upregulation of angiogenesis and ECM organization. Monocytes, macrophages, myeloid dendritic cells, and neutrophils also showed an increasing trend in aCCS, but there was no significant difference. Meanwhile, natural killer cells and CD8 + T cells were decreased in aCCS. The abovementioned results are shown in Fig. 5C and S3F. A heatmap between cell and hub genes (Fig. S3D) indicated that fibroblasts and endothelial cells were correlated with multiple hub genes related to angiogenesis, including *vWF*, *BGN*, *THBD* and *SERPINE1*. These two cell types were also highly correlated, as shown in Fig. S3E.

To further analyze the subpopulation of fibroblasts, single-sample GSEA(ssGSEA) was performed for subpopulation analysis. Lamina propria, SOX6, inflammation and tumor-associated fibroblasts were upregulated, while RSPO3 + fibroblasts were downregulated, and myofibroblasts maintained the same level as control samples (Fig. 6D).

### Estimation and validation via qRT-PCR

**Fig. 7:**
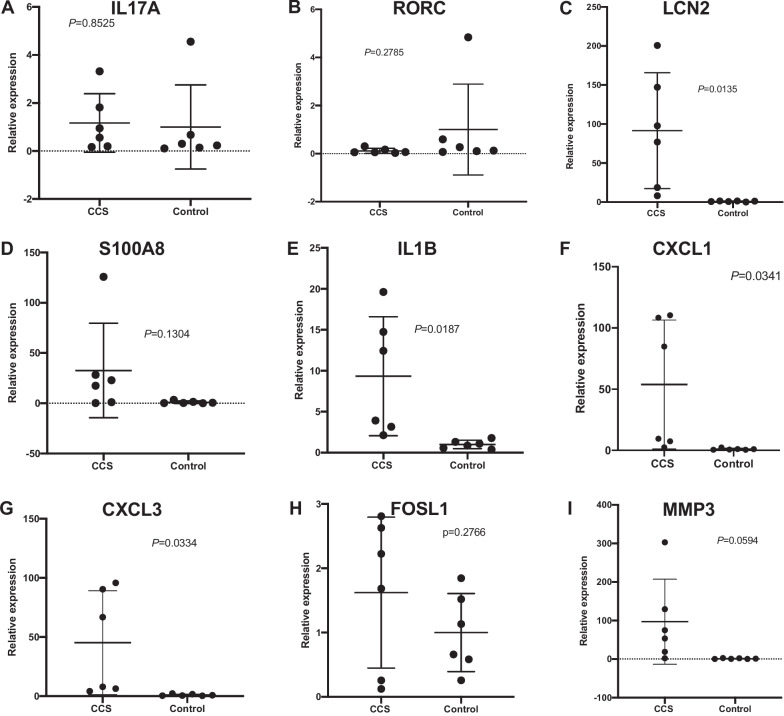
Quantitative real-time PCR results for validation. LCN2, IL1ß, CXCL1, CXCL3 showed significant overexpression in aCCS group. **A** The mRNA expression level of IL17A was not significantly different between the two groups; **B** The mRNA expression level of RORC was not significantly different between the two groups; **C** The mRNA expression level of LCN2 showed significant mRNA overexpression in aCCS group; **D** The mRNA expression level of S100A8 was not significantly different between the two groups; **E** The mRNA expression level of IL1B showed significant mRNA overexpression in aCCS group; **F** The mRNA expression level of CXCL1 showed significant mRNA overexpression in aCCS group; **G** The mRNA expression level of CXCL3 showed significant mRNA overexpression in aCCS group; **H** The mRNA expression level of FOSL1 was not significantly different between the two groups; I. The mRNA expression level of MMP3 was not significantly different between the two groups

To estimate and validate the accuracy of our sequencing data, a subset of genes of the canonical IL-17 signaling pathway, a highly upregulated and representative signaling pathway that plays an important role in other bowel diseases with good drug accessibility [38], was selected for qRT-PCR validation. The validation cohort consisted of samples from six CCS patients and six healthy controls. The mRNA levels of the *LCN2*, *IL1B*, *CXCL1*, and *CXCL3* genes were significantly higher in the CCS patients than in the controls, whereas the mRNA levels of the *IL17A*, *RORC*, *S100A8*, *FOSL1*, and *MMP3* genes were not significantly different between the two groups (Fig. 7). Compared to the differential analysis results based on the count matrix, the concordance was 89% (*FOSL1* was the only gene showing an inconsistent trend with WTS data), reflecting good robustness. In addition, *LCN2*, *IL1B*, *CXCL1*, and *CXCL3* were all hub genes identified in the PPI network analysis.

## Discussion

To our knowledge, this is the first case-control WTS study focusing on active CCS colonic polyps. WTS revealed distinct expression profiles between the colonic hamartomatous polyps of CCS and the colonic mucosa of healthy counterparts. Transcriptomic evidence suggests an involvement of ECM disorganization, inflammatory cell infiltration, increased angiogenesis, and potential EMT, aligning with the morphological features of CCS hamartomatous polyps, including edema, engorgement, hemorrhage, or erosion [2, 3, 5, 7, 8], and revealed possible clues for pathogenesis and treatment modalities.

Aberrant immunity may contribute to the pathophysiological process of colonic hamartomatous polyps in CCS. A speculated autoimmune mechanism was supported by the infiltration in CCS polyps with IgG4 plasma cells and the efficacy of immune modulating therapy [7, 8]. As reported in our previous WES study, germline mutations in genes related to the innate immune response and glycosaminoglycan (known as mucopolysaccharide) binding were prominent in CCS patients [17]. In this WTS analysis, enrichment analyses suggest upregulation of JAK-STAT3, IL-17, cytokine and cytokine receptors, and TNF signaling pathways, reflecting activation of the immune system and especially the innate immune system. We observed elevated expression of genes such as *VWF*, *THBD*, and *SERPINE1*, reflecting dysregulation of the coagulation system and complement system [39]. The general upregulation of C-X-C motif chemokine ligands (*CXCL1*, *CXCL2*, *CXCL3*, *CXCL6*, *CXCL8*) and C-X-C motif chemokine receptors (*CXCR1* and *CXCR2*) implies a potential role for neutrophils and macrophages in CCS. *LCN2* is a classical marker of neutrophils [40], which together with the cyto-typing analysis further supports our hypothesis. *IL-1β*, an identified hub gene in CCS, is a classical target in autoimmune diseases [41]. *IL-1RN* (also known as *IL-1RA*) was also found to be highly expressed. However, the signature genes of the canonical IL-17 pathway, including *IL17A*, *RORC*, *MMP3* and *S100A8*, did not significantly differ between CCS and healthy counterparts, which was consistent with the sequencing results and suggested that canonical IL-17 signaling may not play a vitol role in the colonic pathology of CCS [42]. Transcription factor enrichment analysis identified *RELA* (p65), *NFKB1* (p50/p105) subunit, and *STAT3* as potentially key transcription factors, which may shed light on future therapeutic strategies considering their participation in the TNF-NFκB and IL6-JAK-STAT3 pathways, cell aging and other biological processes [43, 44]. In addition, we observed that VEGF receptors, including *FLT1* (*VEGFR1*) and *KDR* (*VEGFR2*), were significantly upregulated in CCS, which was consistent with Matsumoto Y et al.'s observation of angiogenesis in CCS patients using linked color imaging (LCI) and immunohistochemical confirmation of VEGF overexpression [45]. Integrins are also important players in the biological processes of inflammation, infection and angiogenesis [46]. In our study, *ITGA5*, *ITGA11*, and *ITGB2-AS1* were significantly upregulated, and *ITGA1*, and *ITGB2* were also upregulated (but failed to reach our threshold). *ITGA5*, a hub gene identified in this study, has also been reported to engage in pathological angiogenesis and vascular remodeling [47] and diabetes-related vascular inflammation [48].

The results of cell type enrichment analysis were consistent with the histological features of inflammatory cell infiltration and proliferation potential of CCS hamartomatous polyps. Fibroblasts were massively increased, and further subset analysis revealed a similar pattern to inflammatory bowel disease [35]. Meanwhile, it was observed that cancer-associated fibroblasts were similarly elevated, which may have some association with the upregulation of EMT [49].

Our study provided new clues for gut mucosal barrier dysfunction in CCS [17]. The high prevalence of hypoalbuminemia, elevated fecal a1-antitrypsin clearance and ^99m^Tc-albumin scintigraphy indicated the presence of GI tract protein loss [5]. Morphological observation from an early ultrastructural study suggested that leakage of mucin in CCS patients may be involved in pathogenesis and directly associated with edema [50]. Our previous WES study also found that germline mutations in mucin family genes were frequent in CCS patients [17]. In this study, although most mucin genes did not reach our threshold of |log2 Fold Change|> 2, some mucin genes actually showed a tendency of transcriptional change. For example, the fold changes in *MUC1* and *MUC4* were higher than 1.5, and GSEA found that the mucin-related O-glycosylation pathway was significantly upregulated [17, 51]. In addition, oxidative phosphorylation and the fatty acid metabolism pathway were downregulated in CCS patients, which may be related to impaired metabolism and malnutrition in CCS patients. In addition, downregulated terms were mainly related to disorders of water electrolyte balance and dysfunctional nitrogen balance. Among the downregulated hub genes, *GUCA2A*, *GUCA2B*, *BEST4*, *OTOP2*, *CA7*, *ZG16*, and *BEST2* were representative markers for two classes of recently identified epithelial cells [52], and ssGSEA was performed with credible cell markers. Downregulation of BEST4 cells and goblet cells indicates intestinal epithelial barrier damage and dysregulation of ion transmembrane transport, which may be associated with protein loss-associated hypoalbuminemia [53] and diarrhea [54] in CCS patients [2, 5, 6, 13, 17]. *SLC26A3*, a downregulated hub gene, was previously found to be involved in immunoregulation, intestinal epithelial barrier damage mechanisms and electrolyte homeostasis [55].

The potential for dysplasia and carcinogenesis represents one major challenge in CCS disease management. Despite CCS being regarded as a benign condition, multiple retrospective studies have found a significantly elevated percentage of gastrointestinal neoplasms in CCS, with up to 40% of patients complicated by colonic adenomas [3, 5, 8]. The exact route of carcinogenesis remains unclear. Malignant transformation of a colonic polyp adenoma was noted in multiple independent case reports [11, 56], indicating a possible route from adenoma to carcinoma. In recent years, the hallmark similarities between wound healing and tumor formation have gained much attention, especially with regard to EMT, angiogenesis and inflammatory infiltration [57], which suggests that carcinogenesis may be associated with continuous intestinal injury and inflammatory stimuli. In a large CCS cohort, significantly less cancer development was observed in patients with sustained endoscopic remission than in those who relapsed or failed to respond to therapies, which was similar to the clinical observations on colitis-related colorectal cancer (CRC) in inflammatory bowel diseases [5, 58]. In our study, oncogenic-related pathways, including the Wnt [59] and PI3K-AKT [60] signaling pathways, were significantly upregulated. The IL6-JAK-STAT3 signaling pathway, which plays a vital role in CRC, was also found to be upregulated in this study. In addition, hallmark genes of the TGF-β/Smad pathway, another important CRC pathway, were also found with dysregulation trends in our analysis (*TGF-ß* with an upregulation trend and *SMAD7* with a downregulation trend without reaching the threshold), although the whole pathway was not captured by enrichment analysis [61]. We also observed an upregulation of *CDKN2A* (p16), which is a tumor suppressor gene highly expressed in ulcerative colitis in previous studies. Further investigation is warranted to determine whether CDKN2A plays a similar role as an “emergency brake” in the carcinogenesis of CCS [36, 62]. Overall, the abovementioned findings indicated potentially similar pathological routes between CCS and other disease conditions, such as CRC and HPS-associated malignancies [63]. Preventive measures for CRC- and HPS-related malignancies, including endoscopic mucosal healing and surveillance, may also be applicable to CCS [64, 65].

Compared with previous CCS transcriptome sequencing analysis obtained from gastric polyp tissue [19], our results based on colonic polyps revealed partially consistent expression profiles. *INHBA* mRNA was similarly significantly upregulated in our analysis, and most of the pathway trends were also consistent. Interestingly, protein digestion and absorption, and complement and coagulation cascades showed opposite trends in the two studies, which may reflect the spatially differential characteristics of CCS polyps or may be due to derangements in protein metabolism and the complement system.

Our study has several limitations. CCS is a systemic disease, and CCS colonic polyps are not representative enough to explain the whole spectrum of CCS symptoms. Further omics studies, such as whole genome sequencing, and peripheral blood mononuclear cell-based sequencing or proteomic research, are expected. The rarity of CCS limited the sample size in our study, which restricts further subgroup analysis based on variables such as histology features. It is worth noting that the outlier, CCS5, has different clinical characteristics compared to the other four samples which were included in the final analyses. We cannot exclude the possibility that CCS5 may represent a subgroup of patients with an early onset and poor response to glucocorticoid therapy. Therefore, it would be informative that more samples from this specific type of patients be collected for further study. Not all samples used for RNA sequencing had sufficient remaining cDNA for subsequent qRT-PCR validation, which is one limitation of the study design. However, the results of qRT-PCR are generally consistent with the RNA sequencing, indicating a high level of confidence in the sequencing data. Still, it is necessary to collect more samples for further validation. Further exploration of the mechanisms other than the canonical IL-17 pathway that may be involved in the pathogenesis of CCS is warranted. Considering that CCS has prominent immune involvement, conducting sorted immune cell whole transcriptome sequencing and single-cell sequencing is promising to deepen our understanding of immune mechanisms in CCS. Overall, unraveling the mystery of CCS and improving outcomes for patients with CCS requires concerted efforts in the basic, translational, and clinical fields. Given the rarity of this disease, reliable animal or organoid modeling, in-depth omics studies, and establishing more flexible clinical cohorts should be scheduled.

## Conclusions

In conclusion, our comprehensive case-control whole transcriptome analysis of active CCS hamartomatous colonic polyps has unveiled intricate molecular pathways that may contribute to the pathophysiology of CCS. Our findings underscore the significance of the activated innate immune response, ECM disorganization, inflammatory cell infiltration, increased angiogenesis, and EMT in shaping the disease landscape, which further supports CCS as a chronic inflammatory condition. Meanwhile, the identification of aberrantly activated inflammatory responses in CCS through our research provides rationale for the application of immune-modulating therapies.

The recognition of specific molecular signatures not only enhances the current understanding of CCS pathophysiology but also propels the field toward future breakthroughs in targeted treatment strategies. Future research endeavors may move beyond elucidating the underlying mechanisms, the translational implications of our findings, offering hope for more effective and personalized management for CCS patients.

### Supplementary Information

**Additional file 1. Fig. S1**. Multi-dimensional quality control of this study. A. Correlation plot of 5-paired; B. Hierarchical cluster dendrogram of 5-paired; C. PCA result of 5-paired; D. Correlation plot of 4-paired; E. Hierarchical cluster dendrogram of 4-paired; F. PCA result of 4-paired; G. Disease gene net enrichment result of 4-paired. PCA, principal component analysis.

**Additional file 2. Fig. S2**. Result of GO&KEGG GSEA analyses and Wikipathway & ReactomePA analyses. A. ORA Enrichment result based on upregulated & downregulated genes via ReactomePA database; B. ORA Enrichment result based on upregulated & downregulated genes via Wikipathway database; C. Enrichment result obtained from GSEA analysis via GO; D. Enrichment result obtained from GSEA analysis via KEGG; E. Enrichment result obtained from GSEA analysis via ReactomePA; F. Enrichment result obtained from GSEA analysis via Wikipathway. GSEA, gene set enrichment analysis. ORA, over representation analysis. GO, Gene Ontology. KEGG, Kyoto Encyclopedia of Genes and Genomes.

**Additional file 3: Fig. S3**. Supplementary Results of PPI analyses and Cell Marker Enrichment analyses. A. Correlation plot between hub genes. B. KEGG ORA Enrichment result of based on hub genes. C. GO ORA Enrichment result of based on hub genes. D. Correlation plot showed the correlation between cells and hub genes; E. Correlation plot showed the correlation between different cells; F. Bar plot showed the cell difference between controls and aCCS patients. GO, Gene Ontology. KEGG, Kyoto Encyclopedia of Genes and Genomes. ORA, Over representation analysis. GSVA, Gene Set Variation Analysis. ssGSEA, single sample gene set enrichment analysis, a special type of GSVA. P-value was calculayed by Mann–Whitney U test, * :P-value < 0.05.

**Additional file 4: Table S1**. Baseline characteristics of patients and healthy volunteers in validation group.

**Additional file 5: Table S2**. Primers for Quantitative real-time PCR.

**Additional file 6: Table S3**. Transcription Factor analysis result based on differentially expressed genes (DEGs).

**Additional file 7: Table S4**. Transcription Factor analysis result based on hub genes.

## Data Availability

The genetic data that support the findings of this study are not openly available due to reasons of sensitivity and are available from the corresponding author(liji0235@pumch.cn) upon reasonable request.

## Abbreviations

ADAM: A disintegrin and metalloproteinase domain
CA: Carbonic anhydrase
CCS: Cronkhite‒Canada syndrome
cDNA: Complementary DNA
CRC: Colitis-related colorectal cancer
DEG: Differentially expressed gene
ECM: Extracellular matrix
EMT: Epithelial to mesenchymal transition
FPKM: Fragments per kilobase of exon model per million mapped reads
GI: Gastrointestinal
GO: Gene ontology
GSEA: Gene set enrichment analysis
GSVA: Gene set variation analysis
HCA: Hierarchical cluster analysis
HPS: Hamartomatous polyposis syndrome
HV: Healthy volunteer
KEGG: Kyoto encyclopedia of genes and genomes
LCI: Linked color imaging
MASTER: Molecularly aided stratification for tumor eradication research
MCP: Microenvironment cell populations
MMP: Matrix metalloproteinase
ORA: Overrepresentation analysis
PCA: Principal component analysis
PPI: Protein‒protein interaction
qRT-PCR: Quantitative real-time PCR
SLC: Solute carrier family
ssGSEA: Single-sample GSEA
TF: Transcription factor
TRP: Transient receptor potential
WES: Whole exosome sequencing
WTS: Whole transcriptome sequencing
