# Supplementary figures and images for: Cronkhite‒Canada syndrome as inflammatory hamartomatous polyposis: new evidence from whole transcriptome sequencing of colonic polyps
Source: Orphanet J Rare Dis. 2024 Feb 1;19:35. doi: 10.1186/s13023-024-03038-8 (PMC10832113; doi:10.1186/s13023-024-03038-8)

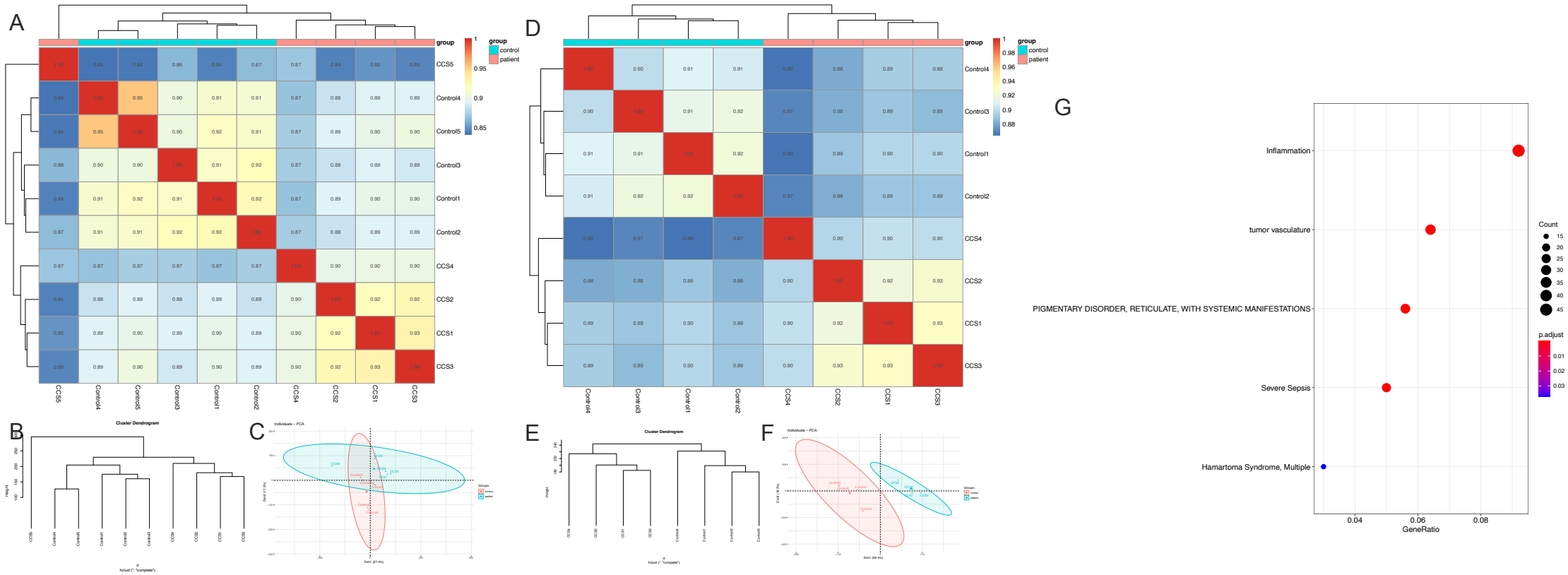

Supplement: Supplementary file 1 — Additional file 1. Fig. S1. Multi-dimensional quality control of this study. A. Correlation plot of 5-paired; B. Hierarchical cluster dendrogram of 5-paired; C. PCA result of 5-paired; D. Correlation plot of 4-paired; E. Hierarchical cluster dendrogram of 4-paired; F. PCA result of 4-paired; G. Disease gene net enrichment result of 4-paired. PCA, principal component analysis. [file 13023_2024_3038_MOESM1_ESM.pdf]

A

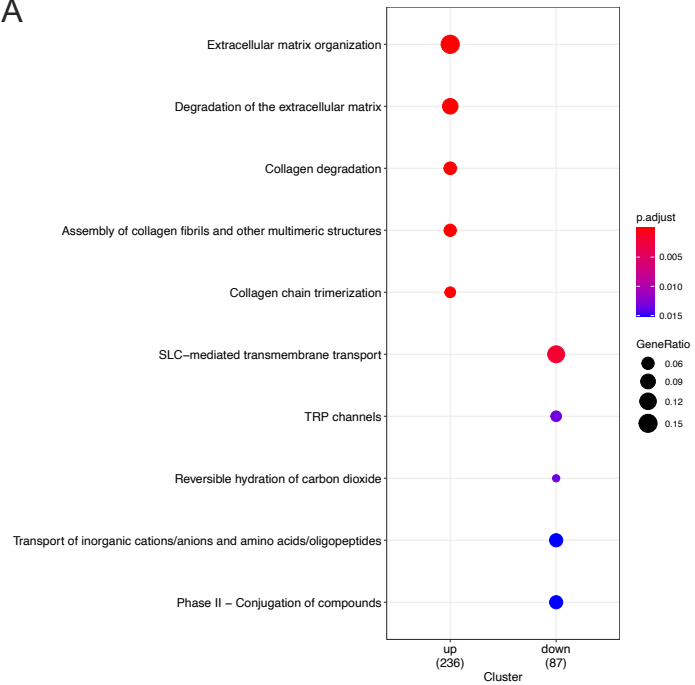

B

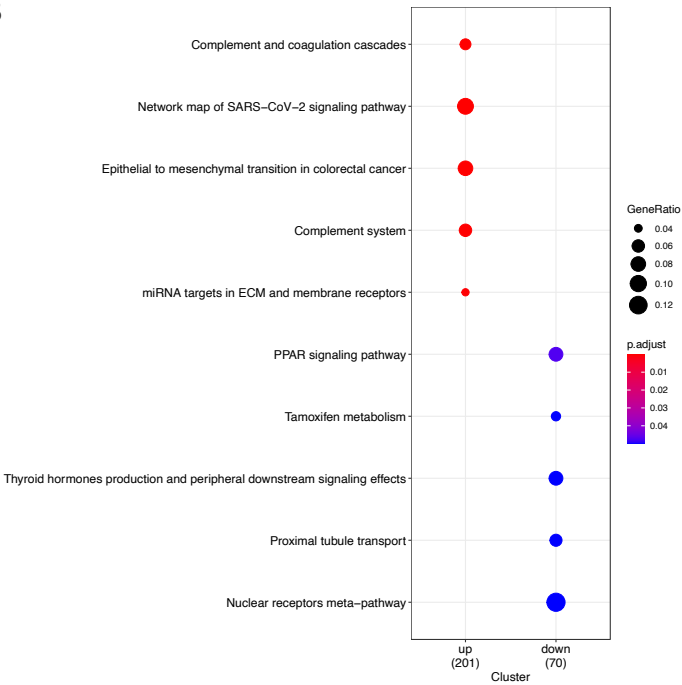

C

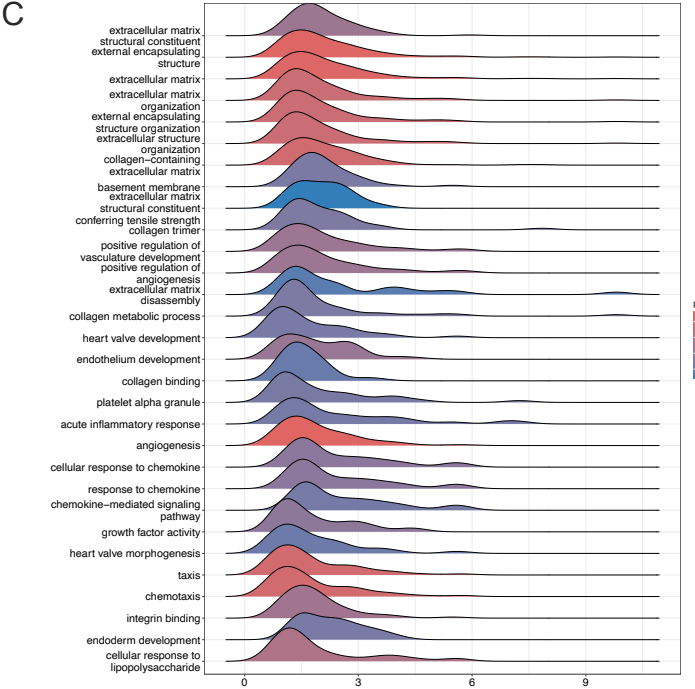

D

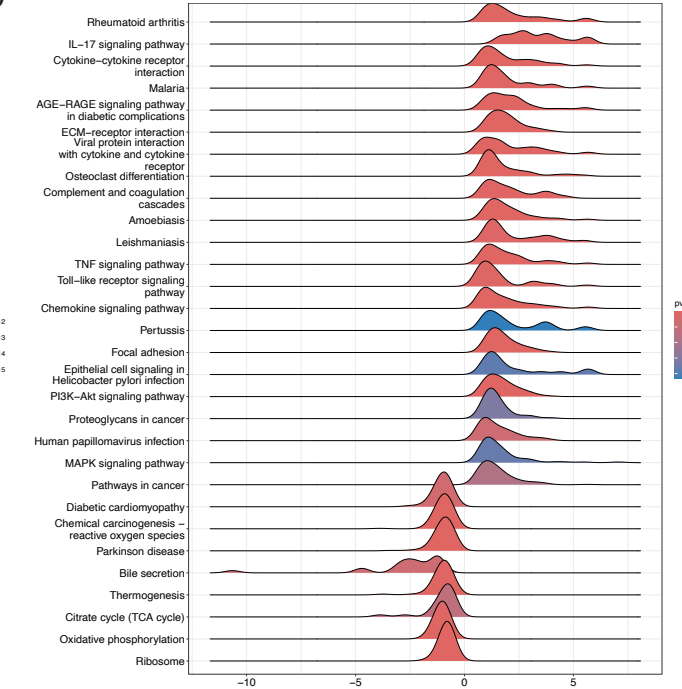

E

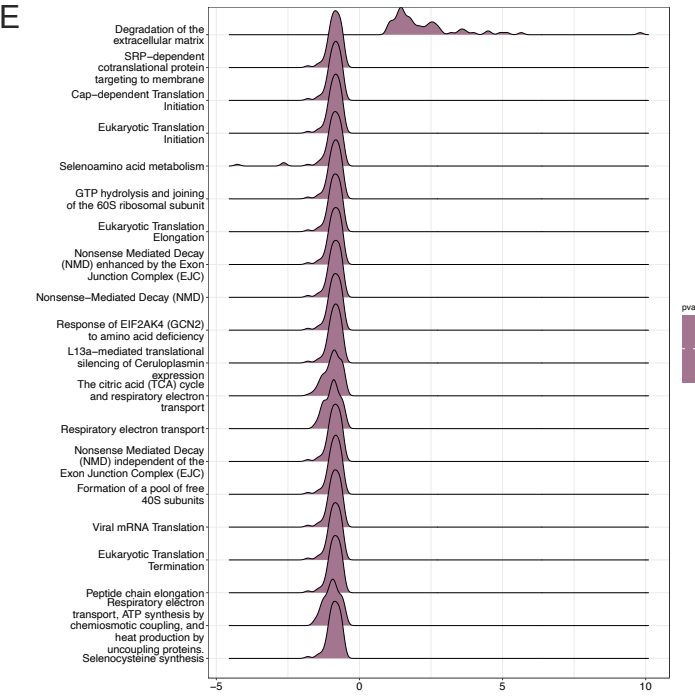

F

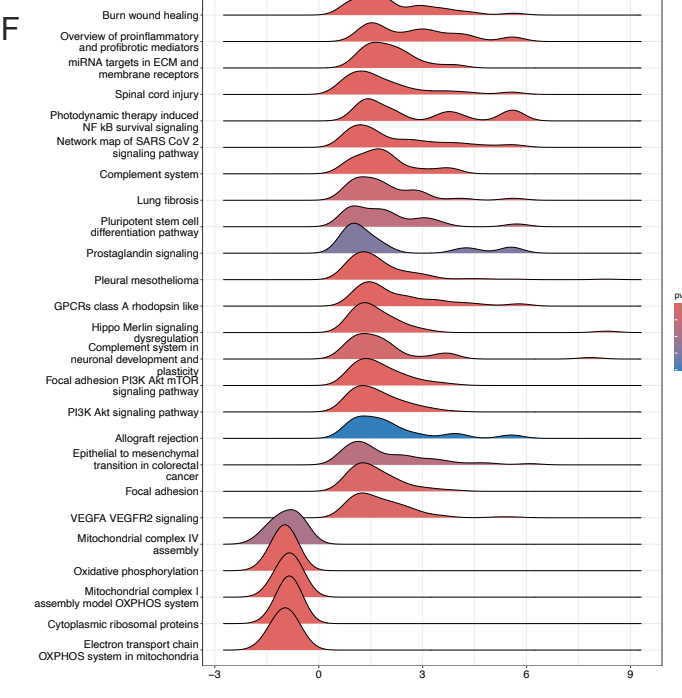

Supplement: Supplementary file 2 — Additional file 2. Fig. S2. Result of GO&KEGG GSEA analyses and Wikipathway & ReactomePA analyses. A. ORA Enrichment result based on upregulated & downregulated genes via ReactomePA database; B. ORA Enrichment result based on upregulated & downregulated genes via Wikipathway database; C. Enrichment result obtained from GSEA analysis via GO; D. Enrichment result obtained from GSEA analysis via KEGG; E. Enrichment result obtained from GSEA analysis via ReactomePA; F. Enrichment result obtained from GSEA analysis via Wikipathway. GSEA, gene set enrichment analysis. ORA, over representation analysis. GO, Gene Ontology. KEGG, Kyoto Encyclopedia of Genes and Genomes. [file 13023_2024_3038_MOESM2_ESM.pdf]

A

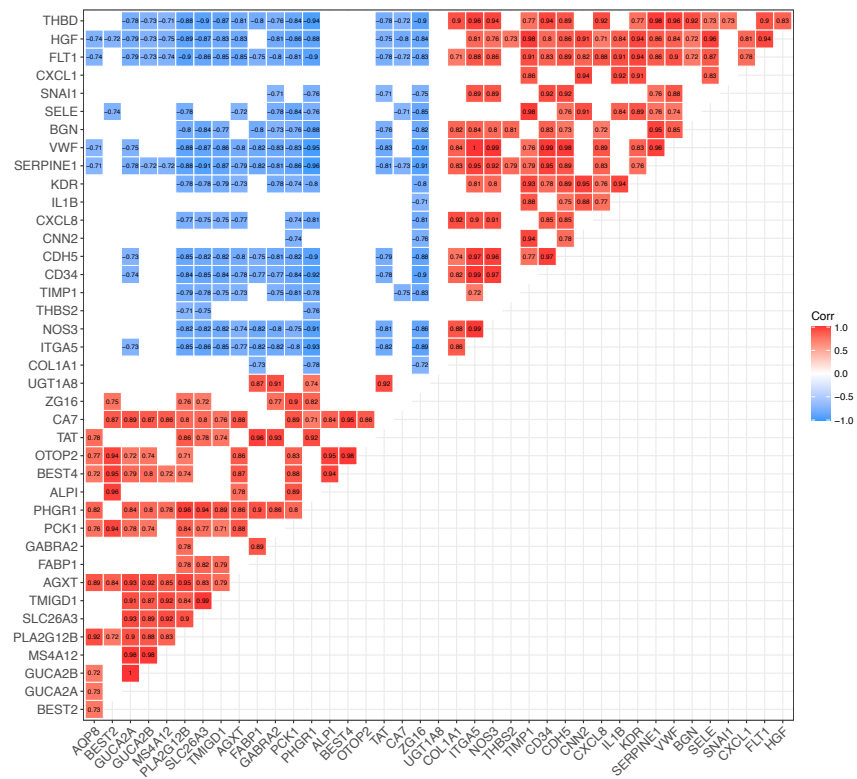

B

AGE-RAGE signaling pathway in diabetic complications

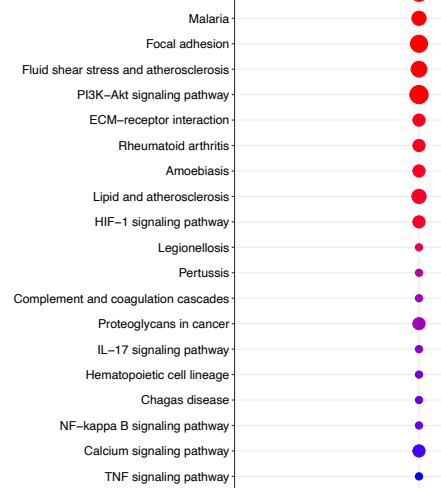

C

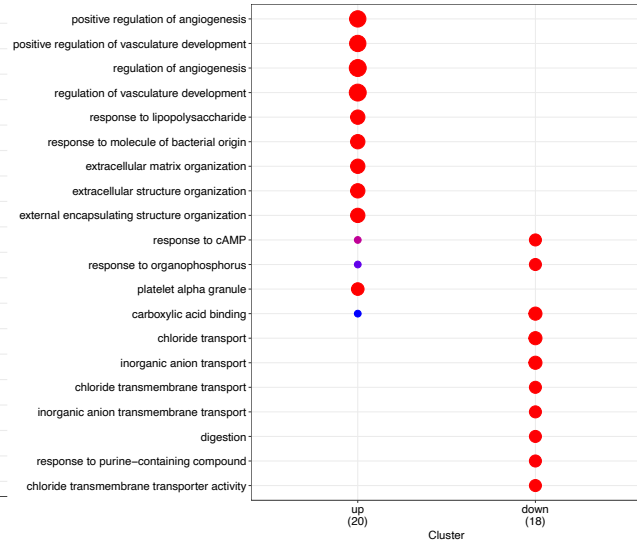

D

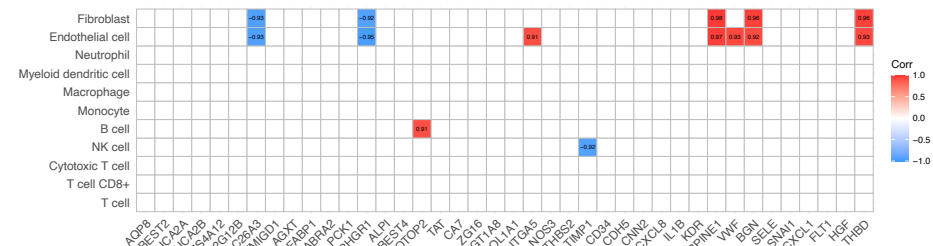

E

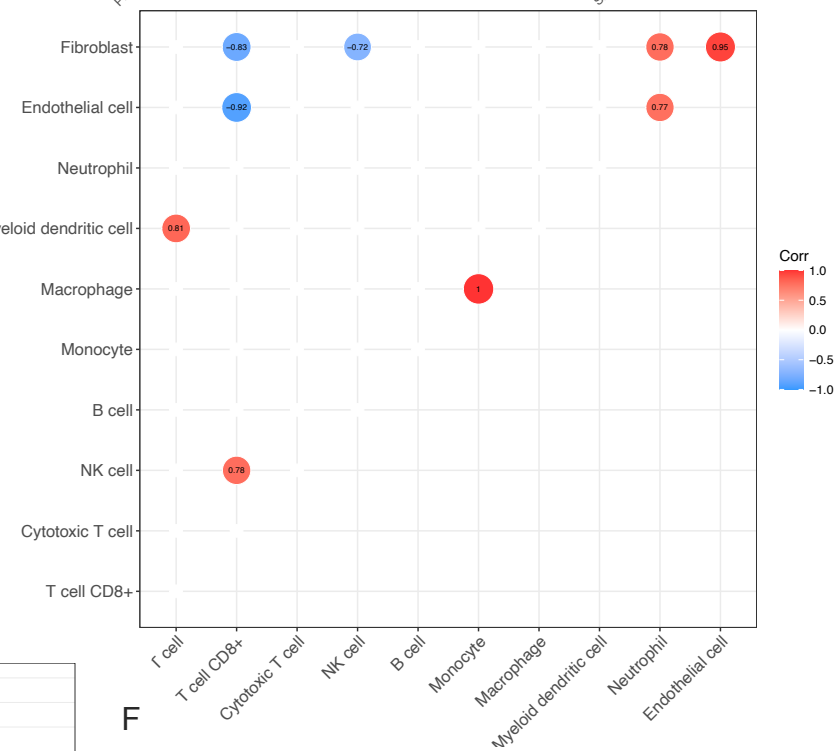

F

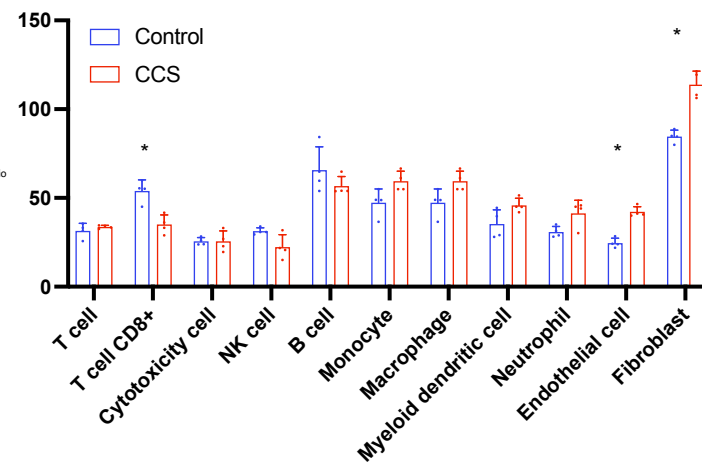

Supplement: Supplementary file 3 — Additional file 3: Fig. S3. Supplementary Results of PPI analyses and Cell Marker Enrichment analyses. A. Correlation plot between hub genes. B. KEGG ORA Enrichment result of based on hub genes. C. GO ORA Enrichment result of based on hub genes. D. Correlation plot showed the correlation between cells and hub genes; E. Correlation plot showed the correlation between different cells; F. Bar plot showed the cell difference between controls and aCCS patients. GO, Gene Ontology. KEGG, Kyoto Encyclopedia of Genes and Genomes. ORA, Over representation analysis. GSVA, Gene Set Variation Analysis. ssGSEA, single sample gene set enrichment analysis, a special type of GSVA. P-value was calculayed by Mann–Whitney U test, * :P-value < 0.05. [file 13023_2024_3038_MOESM3_ESM.pdf]
