# Supplementary material for: Cronkhite‒Canada syndrome as inflammatory hamartomatous polyposis: new evidence from whole transcriptome sequencing of colonic polyps
Source: Orphanet J Rare Dis. 2024 Feb 1;19:35. doi: 10.1186/s13023-024-03038-8 (PMC10832113; doi:10.1186/s13023-024-03038-8)
Supplement: Supplementary file 4 — Additional file 4: Table S1. Baseline characteristics of patients and healthy volunteers in validation group. [file 13023_2024_3038_MOESM4_ESM.docx]

| **pCCS patient** | | | **Control** | | |
| --- | --- | --- | --- | --- | --- |
|  | Age | Gender |  | Age | Gender |
| Patient1 | 63 | Female | Control1 | Female | 53 |
| Patient2 | 56 | Female | Control2 | Female | 61 |
| Patient3 | 61 | Female | Control3 | Female | 67 |
| Patient4 | 61 | Male | Control4 | Male | 48 |
| Patient5 | 46 | Male | Control5 | Male | 60 |
| Patient6 | 68 | Male | Control6 | Male | 48 |

**Table S1 Baseline characteristics of patients and healthy volunteers in validation group**
