# Supplementary material for: Cronkhite‒Canada syndrome as inflammatory hamartomatous polyposis: new evidence from whole transcriptome sequencing of colonic polyps
Source: Orphanet J Rare Dis. 2024 Feb 1;19:35. doi: 10.1186/s13023-024-03038-8 (PMC10832113; doi:10.1186/s13023-024-03038-8)
Supplement: Supplementary file 5 — Additional file 5: Table S2. Primers for Quantitative real-time PCR. [file 13023_2024_3038_MOESM5_ESM.docx]

**Table S2 Primers for Quantitative real-time PCR**

| **mRNA** |  | **Primer Sequence** |
| --- | --- | --- |
| GAPDH | Forward 5’-3’ | GGAGCGAGATCCCTCCAAAAT |
|  | Reverse 5’-3’ | GGCTGTTGTCATACTTCTCATGG |
| IL17A | Forward 5’-3’ | CGGACTGTGATGGTCAACCTGA |
|  | Reverse 5’-3’ | GCACTTTGCCTCCCAGATCACA |
| RORC | Forward 5’-3’ | GAGGAAGTGACTGGCTACCAGA |
|  | Reverse 5’-3’ | GCACAATCTGGTCATTCTGGCAG |
| LCN2 | Forward 5’-3’ | GACAACCAATTCCAGGGGAAG |
|  | Reverse 5’-3’ | GCATACATCTTTTGCGGGTCT |
| S100A8 | Forward 5’-3’ | GGGCATCATGTTGACCGAGC |
|  | Reverse 5’-3’ | GTAACTCAGCTACTCTTTGTGGCTT |
| IL1B | Forward 5’-3’ | ATGATGGCTTATTACAGTGGCAA |
|  | Reverse 5’-3’ | GTCGGAGATTCGTAGCTGGA |
| CXCL1 | Forward 5’-3’ | AGCTTGCCTCAATCCTGCATCC |
|  | Reverse 5’-3’ | TCCTTCAGGAACAGCCACCAGT |
| CXCL3 | Forward 5’-3’ | CGCCCAAACCGAAGTCATAG |
|  | Reverse 5’-3’ | GCTCCCCTTGTTCAGTATCTTTT |
| MMP3 | Forward 5’-3’ | CACTCACAGACCTGACTCGG |
|  | Reverse 5’-3’ | AGTCAGGGGGAGGTCCATAG |
| FOSL1 | Forward 5’-3’ | CAGGCGGAGACTGACAAACTG |
|  | Reverse 5’-3’ | TCCTTCCGGGATTTTGCAGAT |
